# Positive changes in ideal CVH metrics reduce the incidence of stroke

**DOI:** 10.1038/srep19673

**Published:** 2016-01-21

**Authors:** Xiaomeng Yang, Anxin Wang, Xiaoxue Liu, Shasha An, Shuohua Chen, Yilong Wang, Yongjun Wang, Shouling Wu

**Affiliations:** 1 Department of Neurology, Beijing Tiantan Hospital, Capital Medical University, Beijing, China; 2 China National Clinical Research Center for Neurological Diseases, Beijing, China; 3 Center of Stroke, Beijing Institute for Brain Disorders, Beijing, China; 4 Department of Epidemiology and Health Statistics, School of Public Health, Capital Medical University, Beijing, China; 5 Department of Cardiology, Tangshan people’s Hospital, Tangshan, China; 6 Department of Cardiology, Kailuan Hospital, Hebei United University, Tangshan, China

## Abstract

The American Heart Association defined 7 ideal cardiovascular health (CVH) metrics and the benefits of them in reducing the incidence of stroke are well established, but it is unclear whether changes in them alter stroke risk. We calculated the changes of 7 ideal CVH metrics from 2006 to 2008 among 64,373 participants in the Kailuan study. We tested whether changes in the numbers and total scores for the CVH metrics were associated with the incidence of stroke in the 4.89 person-years follow-up. Cox regression modeling was used to estimate the risk of stroke. By year 2008, CVH metrics number of 32.54% participants improved (change ≥+1); 31.90% deteriorated (≤−1); 35.56% stayed the same; In the follow-up,we identified1,182 incident stroke events. Each increase in CVH metrics and every 1-point increase in total CVH score from 2006 to 2008 were associated with reduced odds of total stroke (hazard ratio = 0.87; 95% confidence interval; 0.83–0.92 and 0.89[0.86–0.92] respectively), after adjusting for age, gender, educational level, income and scores for the metrics of ideal CVH at baseline. Positive changes in ideal CVH metrics reduce the incidence of stroke. Our results support the concept that achieving ideal CVH helps to prevent stroke.

In 2010, the American Heart Association (AHA) released its 2020 Impact Goals for cardiovascular health (CVH) promotion and disease reduction^1^. The concept of ideal CVH is defined as the simultaneous presence of 4 ideal health behaviors (not smoking, having a normal body mass index [BMI], being physically active, and eating a healthy diet) and 3 ideal health factors (normal total cholesterol levels, blood pressure [BP], and fasting glucose levels). The presence of more ideal CVH metrics predicts a lower risk of cardiovascular disease (CVD), a lower risk of incident cancer and lower all-cause mortality^2^,^3^,^4^,^5^,^6^. Moreover, several studies have demonstrated the potentially combined protective impact of ideal CVH metrics on the incidence of stroke^7^,^8^,^9^,^10^. It is worthwhile to explore whether changes in health behaviors and factors affect the incidence of CVD and stroke.

Recent studies have demonstrated that changes in ideal CVH status are associated with subclinical atherosclerosis and arterial stiffness, which are potential predictors of CVD and stroke^11^,^12^. Currently, little is known about how well changes in ideal CVH metrics in the follow-up predict the incidence of stroke. Therefore, the present study aim to explore the relationships among changes in ideal CVH metrics and the risk of stroke.

## Results

**Figure 1:**
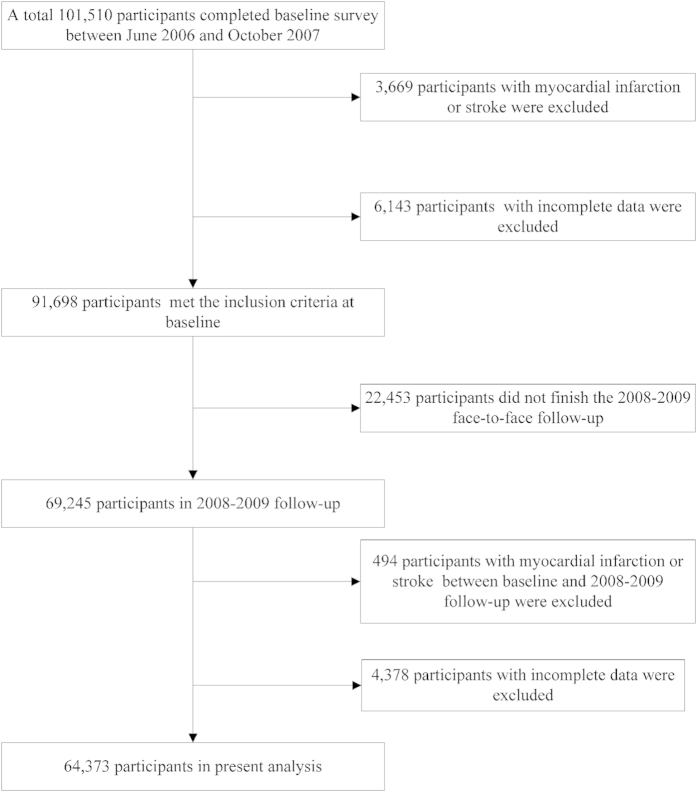
Flowchart of the study.

From June 2006 to October 2007, a total of 101,510 participants (81,110 men and 20,400 women, 18–98 years of age) were recruited to participate in the Kailuan study. At baseline, of the 101,510 participants, we excluded 3,669 with a history of myocardial infarction (MI) or stroke, and 6,143 with incomplete data regarding health factors or health behaviors. As 22,453 participants did not finish the 2008–2009 face-to-face follow-up,the remaining 69,245 participants were examined in 2008–2009. We also excluded 494 participants who had stroke or MI between the baseline and 2008–2009 follow-up and 4,378 with incomplete information. The analyses performed in this study were thus confined to the remaining 64,373 participants (Fig. 1).

### Characteristics at baseline

Characteristics were calculated for the 8 CVH groups (CVH score 0–7) in 2006. Participants with a greater number of ideal CVH metrics were more likely to be women and young, to have attained a higher educational level and to have a higher income (Table 1). The CVH metric scores were inversely related to total cholesterol levels, BP, BMI and fasting glucose levels.

### Changes in CVH metrics and stroke

**Figure 2:**
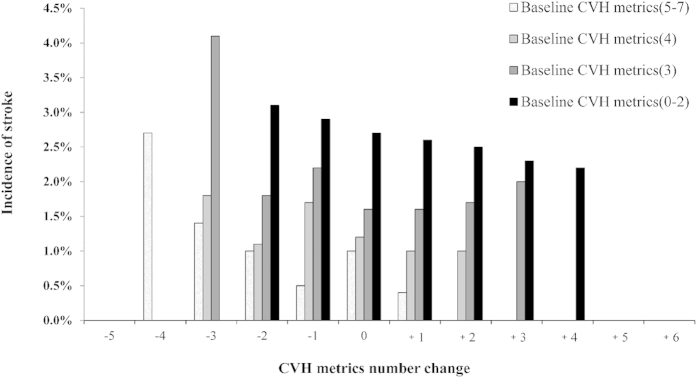
Relationship observed between change of ideal cardiovascular health (CVH) metrics number (from year 2006 to 2008) and the incidence of stroke according to different levels of CVH metrics number at baseline. The horizontal axis indicates the change of CVH metrics number between year 2006 and 2008.

64373 participants were enrolled by year 2008, CVH metrics number of 32.54% participants improved (ideal CVH metrics number change ≥+1); 31.90% deteriorated (change ≤−1); 35.56% stayed the same. The relationship between the CVH metrics number change and the odds of having a stroke was graded; those with a greater increase in the CVH metrics number had a proportionally lower incidence of stroke (Fig. 2).

After adjusting for age, women, education, income, CVH metrics score at baseline, each increase in CVH metrics from 2006 to 2008 was associated with significantly reduced odds of experiencing a stroke (hazard ratios [HR] = 0.87; 95% confidence interval [CI], 0.83–0.92) (Table 2); i.e., such a positive change was associated with a 13% reduction in the odds of experiencing a stroke. Moreover, the interaction between gender and the change in CVH metrics number between 2006 and 2008 was not significant after adjusting for age, educational level, income, and CVH score at baseline (p = 0.727).

Table 3 demonstrates that every 1-point increase in the total score from 2006 to 2008 resulted in a reduction in the odds of having a stroke after adjusting for age, women, education, income, CVH metrics score at baseline (HR = 0.89; 95% CI, 0.86–0.92).

We ran separate models predicting stroke in the follow-up for every 1-point increase of each CVH metric score (Table 4). Every 1-point increase in the total score of physical activity/ total cholesterol/blood pressure/fasting blood glucose from 2006 to 2008 resulted in a reduction in the odds of having a stroke ([HR = 0.91; 95% CI, 0.83–0.99], [HR = 0.84; 95% CI, 0.77–0.92], [HR = 0.59; 95% CI, 0.53–0.66], [HR = 0.81; 95% CI, 0.73–0.89], respectively) after adjusting for age, women, education, income, CVH metrics score at baseline. However, changes in total score of smoke, salt intake and BMI were not significantly associated with the occurrence of stroke in the follow-up.

To determine whether any single CVH metric accounted for the association between the combined CVH score and stroke, we removed each CVH metric one at a time and then reexamined the association (Table 5). The residual composite CVH metric remained significantly associated with stroke.

## Discussion

Positive changes in ideal CVH metrics numbers and in overall CVH scores were significantly associated with a lower risk of stroke. These associations remained after adjusting for demographic variables and for baseline values for CVH metrics. To our knowledge, this is the first study to explore the relationship between changes in CVH metrics and the incidence of stroke. The Northern Manhattan Study (NOMAS)^8^ previously showed that an increase in the number of ideal health factors was associated with a reduced risk of stroke. Kulshreshtha^9^ *et al.* expanded on these findings by considering the full range of poor, intermediate, and ideal scores. These authors demonstrated that an improvement in one component of an ideal health metric by one level (e.g., from poor to intermediate or from intermediate to ideal) was associated with an 8% lower risk of stroke. Zhang^10^ *et al.* further demonstrated the potentially combined protective impact of ideal CVH metrics on ischemic and intracerebral hemorrhagic stroke in a Chinese population. Moreover, several studies^7^,^13^,^14^,^15^ have demonstrated a combined effect of several healthy lifestyle factors on stroke risk, which indicates that changes in lifestyle may reduce the incidence of stroke.

Previous studies have assessed this association at a single time point. Our findings demonstrate the benefits of increasing the number of ideal CVH indicators and of improving CVH score levels on the risk of stroke during the follow-up period. Furthermore, the effectiveness persisted when any 1 CVH metric was omitted, indicating the utility of the 7 CVH metrics as a whole. Because shifts in the distribution of risk factors within a population can have a dramatic impact on reducing the burden of disease, clinicians can help to reduce the risk of stroke in their patient populations by helping patients to maximize the number of ideal CVH metrics and by helping patients with poor health to transition to intermediate or ideal health.

Recent studies have suggested possible mechanisms responsible for reductions in the risk of stroke. Spring^12^ *et al.* observed that alterations in health behaviors are linked to alterations in the burden of subclinical atherosclerosis (coronary artery calcification and carotid intima-media thickness) after adjusting for demographic variables, medications, and baseline health and life factors. Because previous studies^16^,^17^,^18^ have demonstrated that carotid intima-media thickness is predictive of incident clinical stroke, it is reasonable to hypothesize that healthier behaviors, together with lower intima-media thickness, may contribute to a lower risk of stroke. In addition, Aatola^11^ *et al.* demonstrated that changes in ideal CVH status (both from childhood to adulthood and from young adulthood to middle age) were independent predictors of adult pulse-wave velocity (PWV), which is an index of arterial stiffness. A significant association between PWV and cerebral small vessel disease (SVD) has already been reported in several studies^19^,^20^; PWV exposes the small vessels in the brain to highly pulsatile pressure and flow, which may contribute to the pathogenesis of cerebral SVD. The consequences of SVD for the brain parenchyma are primarily lesions, such as lacunar infarcts, white matter lesions, large hemorrhages, and microbleeds, in subcortical structures^21^. In our study, we explored the relationship between changes in ideal CVH metrics and cerebrovascular disease, including ischemic and hemorrhagic stroke. It will be of great value to determine the association between changes in ideal CVH metrics and the incidence of cerebral SVD.

Consistent with previous studies^9^,^15^,^22^ that have demonstrated significant associations between ideal health behaviors or factors and the risk of total stroke, we also observed favorable effects of increased numbers of ideal CVH metrics on the incidence of total stroke. Although some studies^7^,^10^ have also observed a graded, inverse association between the number of healthy lifestyle indicators and the risk of ischemic and/or hemorrhagic stroke, the separate effects on ischemic or hemorrhagic stroke were not significant in our study. A possible reason for this difference may be that the relatively lower incidence of stroke that we observed precluded the association from reaching significance when stroke subtypes were considered individually.

Our study has several strengths, and it demonstrated, for the first time, the relationship between changes in CVH metrics and the incidence of stroke, with consecutive follow-up examinations, validated diagnostic methods and rigorous ascertainment of stroke outcomes. However, some limitations to our results should be considered. First, the Kailuan study was not a nationwide study, and a large proportion of the participants were manual workers. Thus, our findings may not be directly generalizable to other Chinese populations. Second, we used modified definitions of physical activity and diet to compute scores for the relevant CVH metrics. Salt intake is consistently associated with a risk of stroke^23^, and excessive intake of salted food is a serious problem in China. Moreover, the assessments of diet, smoking habits, and physical activity levels were all based on self-reports; thus, these assessments may not provide fully accurate information about exposure levels. Third, the mean follow-up period may not have been long enough to observe a sufficiently large number of stroke events to be able to classify these events into different subtypes for further discussion. Finally, we cannot completely exclude the effects of residual confounding factors that may be related to unmeasured lifestyle factors, such as socioeconomic or neuropsychological covariates (e.g., stress and depression), that may be associated with stroke risk.

In conclusion, our study demonstrates the favorable effect of positive changes in the number of ideal CVH metrics and in the levels of CVH scores on the risk of stroke in a Chinese population. Our findings provide evidence in support of the importance of promoting ideal health behaviors and factors for preventing stroke. Future prospective studies with larger sample sizes, longer follow-up periods and more detailed imaging are needed to determine whether our results hold in larger populations and over longer time periods and to explore the potential relationships between changes in CVH metrics and stroke subtypes.

## Methods

### Study Design and Population

The Kailuan study^4^ was a prospective cohort study conducted in the community of Kailuan in Tangshan, which is a large, modern city southeast of Beijing.

All participants underwent questionnaire assessments and clinical and laboratory examinations conducted in the 11 hospitals responsible for healthcare of this community. Measurements of parameters were made biennially, and we recorded adverse events annually. The study was performed in accordance with the guidelines of the Helsinki Declaration and was approved by the Ethics Committees of the Kailuan General Hospital, the Beijing Chaoyang Hospital, and the Beijing Tiantan Hospital. Every participant will sign a informed-consent before his/her participantion.

### Ideal CVH metrics

Compared with the concept of ideal CVH in 2010^1^, we used alternative measures for the dietary and physical activity metrics in this study. Ideal health behaviors and factors (no history of smoking, consumption of salt <6 g/d, moderate or vigorous physical activity for >80 min per week, BMI <25 kg/m^2^, untreated systolic BP <120 mmHg and diastolic BP <80 mmHg, untreated fasting blood glucose level <100 mg/dL, and untreated total cholesterol level <200 mg/dL) were defined and described in our previous study^10^.

In accordance with AHA definitions, scores for the 7 CVH metrics were classified as ideal, intermediate, or poor. We generated a total score that ranged from 0 to 14 by assigning a value of 0 for poor health, 1 for intermediate health, and 2 for ideal health for each metric. Demographic data (age, sex, household income and educational level) were collected using questionnaires.

### Changes in number and total score of 7 ideal CVH metrics

Changes in the number of ideal CVH metrics (range −7 to +7) and in the total score (range −14 to +14) were calculated by subtracting the number or total score for the metrics obtained in 2006 from the number or total score obtained in 2008.

### Follow-up and Stroke Assessment

The participants were followed-up in face-to-face interviews conducted at every 2-year routine medical examination until December 31, 2013 or to the event of interest, or death. The follow-ups were performed by trained physicians who were blinded to the baseline data that have been described in our previous study^10^. The primary outcome was the first occurrence of stroke, which was either the first nonfatal stroke or a stroke that resulted in death without a preceding nonfatal event. Stroke was diagnosed according to World Health Organization criteria^24^, and brain computed tomography (CT) or magnetic resonance (MR) scans were performed to confirm diagnoses. Strokes were classified into 3 main types: cerebral infarction, intracerebral hemorrhage, and subarachnoid hemorrhage. All stroke outcomes were validated by the Data Safety Monitoring Board and by the Arbitration Committee for Clinical Outcomes. Because of the small number of patients with subarachnoid hemorrhage (n = 33) and the differing etiologies of this type of stroke, we did not include subarachnoid hemorrhage in this study.

### Statistical Analyses

Continuous variables were described as means ± SD and were compared using ANOVAs. Categorical variables were described as percentages and were compared using χ^2^ tests. For trends, we assigned a numeric value to the number of ideal CVH metrics for each subject and analyzed this value as a continuous variable.

Cox proportional hazards regression was performed with changes in the number and total scores of CVH metrics as the covariate, to obtain the hazard ratios (HR) and two-sided 95% confidence intervals (CI) of each increase in CVH metrics and every 1-point increase in total CVH score for the primary outcome of the first occurrence of stroke. In the model, we adjusted for age, gender, educational level and the average monthly income of each family member.

Because 11 hospitals participated in the study, we used a Cox proportional hazards model with a sandwich covariance matrix as a random effect to account for the potentially confounding effect of multiple hospitals participating in the study. All statistical tests were 2-sided, and the significance level was set at 0.05. Statistical analyses were performed using SAS 9.3 (SAS Institute; Cary, NC).

## Additional Information

**How to cite this article**: Yang, X. *et al.* Positive changes in ideal CVH metrics reduce the incidence of stroke. *Sci. Rep.* **6**, 19673; doi: 10.1038/srep19673 (2016).

## Figures and Tables

**Table 1:** Baseline characteristics of participants by number of CVH metrics in 2006.

Variable	Number of Ideal CVH Metrics in 2006								
	0	1	2	3	4	5	6	7	P Value
No. of participants	939	5795	14882	20700	15259	5859	860	79	<0.001
Age, year, mean (SD)	50.88 ± 8.83	50.66 ± 10.27	51.38 ± 10.93	50.87 ± 11.72	49.77 ± 12.77	46.43 ± 13.64	47.5 ± 14.48	51 ± 12.23	<0.001
Women, n (%)	11 (1.2)	460 (7.9)	1979 (13.3)	3927 (19.0)	4345 (28.5)	2776 (47.4)	439 (51.0)	41 (51.9)	<0.001
High school or above, n (%)	173 (18.4)	1134 (19.6)	2770 (18.6)	3955 (19.1)	3557 (23.3)	2167 (37.0)	410 (47.7)	37 (46.8)	<0.001
Income > 800, RMB/month, n (%)	153 (16.3)	975 (16.8)	2152 (14.5)	2773 (13.4)	2182 (14.3)	1177 (20.1)	219 (25.5)	16 (20.3)	<0.001
BMI, kg/m^2^, mean (SD)	28.22 ± 2.54	27.6 ± 2.83	26.63 ± 3.17	25.24 ± 3.36	23.37 ± 2.86	22.33 ± 2.39	22.04 ± ± 2.11	21.79 ± 2.08	<0.001
TC,mmol/L, mean (SD)	235.86 ± 37.56	224.77 ± 42.05	206.52 ± 45.94	187.76 ± 43.36	176.53 ± 37.29	170.26 ± 30.87	167.12 ± 26.95	169.81 ± 20.17	<0.001
SBP, mmHg, mean (SD)	140.94 ± 20.75	138.31 ± 18.81	135.52 ± 18.93	131.67 ± 19.24	124.28 ± 19.27	112.53 ± 16.74	111.77 ± 16.26	105.99 ± 8.79	<0.001
DSP, mmHg, mean (SD)	90.24 ± 11.87	88.76 ± 10.88	86.81 ± 10.75	84.34 ± 10.76	79.86 ± 10.69	72.80 ± 9.36	72.48 ± 8.70	69.93 ± 6.15	<0.001
FBG, mmol/L, mean (SD)	143.45 ± 45.78	119.57 ± 41.56	104.87 ± 34.41	95.33 ± 23.89	90.89 ± 16.58	88.8 ± 12.98	87.89 ± 10.05	86.55 ± 9.97	<0.001

Data are presented as n, n (%) or mean ± SD. ANOVAs and χ^2^ tests were performed.

CVH indicates cardiovascular health; TC, total cholesterol; SBP, systolic blood pressure; DSP, diastolic blood pressure; FBG, fasting blood glucose; BMI, body mass index.

**Table 2:** Change in CVH metrics number from 2006 to 2008 predicting stroke in the follow-up.

Variable	Stroke (n = 1182)		Ischemic (n = 978)		Intracerebral hemorrhagic (n = 196)	
	HR (95% CI)^a^	P Value	HR (95% CI)^b^	P Value	HR (95% CI)^c^	P Value
Model 1	0.89 (0.85–0.94)	<0.001	0.87 (0.82–0.92)	<0.001	0.99 (0.87–1.13)	0.87
Model 2	0.87 (0.83–0.92)	<0.001	0.85 (0.80–0.90)	<0.001	0.97 (0.85–1.11)	0.67
Model 3	0.87 (0.83–0.92)	<0.001	0.85 (0.80–0.90)	<0.001	0.97 (0.85–1.11)	0.67

CVH indicates cardiovascular health; HR, hazard ratios; CI, confidence interval; Cox proportional hazards regression was performed.

^a^Model 1: Adjusted for CVH metrics score at baseline.

^b^Model 2: Adjusted for age, women, CVH metrics score at baseline.

^c^Model 3: Adjusted for age, women, education, income, CVH metrics score at baseline.

**Table 3:** Change in CVH metrics score from 2006 to 2008 predicting stroke in the follow-up.

Variable	Stroke (n = 1182)		Ischemic (n = 978)		Intracerebral hemorrhagic (n = 196)	
	HR (95% CI)^a^	P Value	HR (95% CI)^b^	P Value	HR (95% CI)^c^	P Value
Model 1	0.91 (0.88–0.94)	<0.001	0.90 (0.87–0.94)	<0.001	0.93 (0.85–1.00)	0.06
Model 2	0.89 (0.86–0.92)	<0.001	0.88 (0.85–0.92)	<0.001	0.91 (0.84–0.99)	0.03
Model 3	0.89 (0.86–0.92)	<0.001	0.88 (0.85–0.92)	<0.001	0.91 (0.84–0.99)	0.03

CVH indicates cardiovascular health; HR, hazard ratios; CI, confidence interval; Cox proportional hazards regression was performed.

^a^Model 1: Adjusted for CVH metrics score at baseline.

^b^Model 2: Adjusted for age, women, CVH metrics score at baseline.

^c^Model 3: Adjusted for age, women, education, income, CVH metrics score at baseline.

**Table 4:** Change in each CVH metric score from 2006 to 2008 predicting stroke in the follow-up.

CVH metric	Stroke (n = 1182)		Ischemic (n = 978)		Intracerebral hemorrhagic (n = 196)	
	HR (95% CI)^a^	P Value	HR (95% CI)^a^	P Value	HR (95% CI)^a^	P Value
Smoke	1.00 (0.92–1.07)	0.89	0.97 (0.89–1.05)	0.45	1.17 (0.97–1.41)	0.11
Salt intake	1.06 (0.94–1.19)	0.33	1.03 (0.91–1.17)	0.60	1.14 (0.86–1.50)	0.38
Physical activity	0.91 (0.83–0.99)	0.045	0.93 (0.84–1.03)	0.15	0.84 (0.67–1.05)	0.12
TC	0.84 (0.77–0.92)	<0.001	0.82 (0.75–0.91)	<0.001	0.92 (0.73–1.15)	0.45
BP	0.59 (0.53–0.66)	<0.001	0.61 (0.54–0.69)	<0.001	0.52 (0.39–0.69)	<0.001
FBG	0.81 (0.73–0.89)	<0.001	0.79 (0.71–0.88)	<0.001	0.83 (0.65–1.06)	0.14
BMI	0.89 (0.79–1.01)	0.07	0.90 (0.79–1.03)	0.14	0.86 (0.64–1.16)	0.31

CVH indicates cardiovascular health; HR, hazard ratios; CI, confidence interval; TC, total cholesterol; BP, blood pressure; FBG, fasting blood glucose ; BMI, body mass index; Cox proportional hazards regression was performed.

^a^Adjusted for age, gender, education, income, CVH score at baseline.

**Table 5:** Hazard ratios (P Value) for CVH metrics score change when 1 CVH metric is omitted.

Omitted CVH metric	Stroke (n = 1182)		Ischemic (n = 978)		Intracerebral hemorrhagic (n = 196)	
	HR (95% CI)^a^	P Value	HR (95% CI)^a^	P Value	HR (95% CI)^a^	P Value
Smoke	0.87 (0.84–0.90)	<0.001	0.87 (0.83–0.91)	<0.001	0.87 (0.79–0.95)	0.002
Salt intake	0.87 (0.84–0.90)	<0.001	0.87 (0.83–0.90)	<0.001	0.89 (0.82–0.91)	0.01
Physical activity	0.89 (0.86–0.92)	<0.001	0.88 (0.85–0.92)	<0.001	0.92 (0.85–1.02)	0.11
TC	0.89 (0.85–0.92)	<0.001	0.88 (0.85–0.92)	<0.001	0.90 (0.83–0.99)	0.03
BP	0.91 (0.88–0.95)	<0.001	0.90 (0.87–0.94)	<0.001	0.95 (0.87–1.05)	0.32
FBG	0.90 (0.86–0.93)	<0.001	0.89 (0.86–0.93)	<0.001	0.92 (0.84–1.01)	0.07
BMI	0.88 (0.85–0.91)	<0.001	0.87 (0.84–0.91)	<0.001	0.91 (0.83–0.99)	0.03

CVH indicates cardiovascular health; HR, hazard ratios; CI, confidence interval; TC, total cholesterol; BP, blood pressure; FBG, fasting blood glucose; BMI, body mass index; Cox proportional hazards regression was performed.

^a^Adjusted for age, gender, education, income, CVH score at baseline and 6 indicator variables representing the 7 possible baseline CVH metrics.
